# Enhanced Electrocatalytic Nitrate Reduction to Ammonia Using Functionalized Multi-Walled Carbon Nanotube-Supported Cobalt Catalyst

**DOI:** 10.3390/nano14010102

**Published:** 2024-01-01

**Authors:** Minghao Ye, Xiaoli Jiang, Yagang Zhang, Yang Liu, Yanxia Liu, Lin Zhao

**Affiliations:** 1 School of Materials and Energy, University of Electronic Science and Technology of China, Chengdu 611731, China; 202221210139@std.uestc.edu.cn (M.Y.); jiangxl@std.uestc.edu.cn (X.J.); 202221030136@std.uestc.edu.cn (Y.L.); liuyanxia100@uestc.edu.cn (Y.L.); zhaolin316@uestc.edu.cn (L.Z.); 2 State Key Laboratory of Electronic Thin Films and Integrated Devices, University of Electronic Science and Technology of China, Chengdu 611731, China

**Keywords:** nitrate reduction, electrochemical active surface area, electrocatalysts, multi-walled carbon nanotubes

## Abstract

Ammonia (NH_3_) is vital in modern agriculture and industry as a potential energy carrier. The electrocatalytic reduction of nitrate (NO_3_^−^) to ammonia under ambient conditions offers a sustainable alternative to the energy-intensive Haber−Bosch process. However, achieving high selectivity in this conversion poses significant challenges due to the multi-step electron and proton transfer processes and the low proton adsorption capacity of transition metal electrocatalysts. Herein, we introduce a novel approach by employing functionalized multi-walled carbon nanotubes (MWCNTs) as carriers for active cobalt catalysts. The exceptional conductivity of MWCNTs significantly reduces charge transfer resistance. Their unique hollow structure increases the electrochemical active surface area of the electrocatalyst. Additionally, the one-dimensional hollow tube structure and graphite-like layers within MWCNTs enhance adsorption properties, thus mitigating the diffusion of intermediate and stabilizing active cobalt species during nitrate reduction reaction (NitRR). Using the MWCNT-supported cobalt catalyst, we achieved a notable NH_3_ yield rate of 4.03 mg h^−1^ cm^−2^ and a high Faradaic efficiency of 84.72% in 0.1 M KOH with 0.1 M NO_3_^−^. This study demonstrates the potential of MWCNTs as advanced carriers in constructing electrocatalysts for efficient nitrate reduction.

## 1. Introduction

Ammonia, a versatile chemical compound, has essential applications in a wide range of industries including agriculture, pharmaceuticals, metallurgy, explosives, and textiles, and as a crucial precursor for fertilizer production [1,2,3,4]. Conventionally, ammonia is produced through the Haber–Bosch (H-B) process, which involves the synthesis of ammonia by combining nitrogen and hydrogen gas under high temperature and pressure [5]. However, this traditional Haber-Bosch process is known for significant energy consumption and relatively low overall efficiency [6]. Recent studies have explored alternative methods for ammonia (NH_3_) synthesis, particularly using electrochemical methods under mild conditions [7,8]. Unfortunately, the poor solubility of N_2_ in H_2_O and the high energy barrier associated with breaking the N≡N bond (941 kJ mol^−1^) present significant challenges for electrochemical nitrogen reduction (NRR) with a high yield of NH_3_ [9]. Compared with highly stable N_2_ molecules, nitrate ion (NO_3_^−^) may be a better nitrogen source for large-scale electrochemical synthesis of NH_3_ [10]. The high solubility of NO_3_^−^ in water and the low dissociation energy of N=O bonds (204 kJ mol^−1^) promote the reaction kinetics of NH_3_ generation [11,12]. In addition, the excessive use of nitrogen fertilizers and fuel combustion have made NO_3_^−^ one of the most common water pollutants, leading to the deterioration of water quality and posing a threat to human health [13]. In this context, electrochemical reduction of aqueous nitrate to ammonia could mitigate nitrate pollution in water bodies while simultaneously supplementing ammonia production without relying on fossil fuels. The electrocatalytic reduction of nitrate involves complicated processes with transfer of multi-electrons and protons, leading to the production of various species, such as NH_2_OH, NH_3_, NO, and NO_2_^−^ (see Equations (1)–(5)) [14].

(1) $${{\mathrm{NO}}_{3}}^{-}+2e^{-}+H_{2}O\;⇌\;{{\mathrm{NO}}_{2}}^{-}\;+\;{2\mathrm{OH}}^{-}$$

(2) $${{\mathrm{NO}}_{2}}^{-}+H_{2}O+e^{-}\;⇌\;\mathrm{NO}+{2\mathrm{OH}}^{-}$$

(3) $$\mathrm{NO}+e^{-}\;⇌\;{\mathrm{NO}}^{-}$$

(4) $${\mathrm{NO}}^{-}+2e^{-}+H_{2}O\;⇌\;{\mathrm{NH}}_{2}\mathrm{OH}\;+\;{\mathrm{OH}}^{-}$$

(5) $${\mathrm{NH}}_{2}{\mathrm{OH}}^{-}+2e^{-}+H_{2}O\;⇌\;{\mathrm{NH}}_{3}\;+\;{\mathrm{OH}}^{-}$$

Currently, electrocatalysts based on noble metals, such as Rh, Ru, and Ir, have shown excellent performance in electrocatalytic reduction of nitrate to produce ammonia, achieving high Faradaic efficiency even at low overpotentials [15,16,17,18]. However, when subjected to more negative potentials, these noble metal electrocatalysts are susceptible to the competing hydrogen evolution reaction (HER) due to their high proton affinity. This trend becomes especially prominent in electrolytes with low nitrate concentrations, leading to reduced yields and selectivity for nitrate reduction [19,20]. Moreover, the high cost and limited global reserves of precious metals severely constrain their application and commercialization. Therefore, the pursuit of non-noble metal electrocatalysts as promising candidates for nitrate electroreduction has gained significant interest.

Non-noble metallic electrocatalysts, such as Cu, Co, Ti, Ni, and Fe, have been studied for their potential in the electrochemical reduction of nitrate in water. Among these, copper and cobalt-based catalysts are considered the most promising non-precious metal-based electrocatalysts owing to their low cost, high activity, and large-scale production potential [2,21,22]. Previous works demonstrated that transition metals (such as cobalt) possess stronger nitrogen oxide adsorption and catalytic activities [23,24]. However, the utilization of pristine cobalt-based electrocatalysts encounters challenges associated with low conductivity and proton affinity. These drawbacks lower the yield and Faraday efficiency in electrocatalytic reduction of nitrate. It is well-known that multi-walled carbon nanotubes are excellent conductors and possess a high specific surface area, and can serve as ideal supports to enhance the activity and efficiency of the electrocatalyst [25,26,27,28,29].

Bearing this in mind, we prepared a uniformly dispersed Co/MWCNTs catalyst via a straightforward wet chemical reduction method in an aqueous solution. The catalyst demonstrates outstanding catalytic performance, achieving a remarkable Faradaic efficiency of 84.72% for NH_3_ production under alkaline conditions at −0.16 V vs. RHE. Electrochemical test results revealed that the electrochemical activity of the Co/MWCNTs catalyst surpassed that of the Co/carbon powder. This enhancement can be attributed to the effective conductivity improvement imparted by the multi-walled carbon nanotubes, thereby facilitating mass-charge transport within the electrocatalyst. Simultaneously, it provides a larger active surface area, enabling a higher exposure of accessible active cobalt sites to interact with nitrate ions in the electrolyte. This work provides a new path for improving the reduction ability of non-noble metal electrocatalysts in the ammonia production process.

## 2. Materials and Methods

### 2.1. Materials

Cobalt nitrate hexahydrate (Co(NO_3_)_2_·6H_2_O, 99%) was purchased from Jiangsu Aikang Biomedical R&D Co., Ltd. (Nanjing, China). Multi-walled carbon nanotubes were purchased from Tianjin Crystal New Material Technology Co., Ltd. (Tianjin, China). Ethanol (CH_3_CH_2_OH, 99%) was obtained from Chengdu Keweidzhuo Technology Co., Ltd. (Chengdu, China). Carbon paper and Nafion membrane were acquired from Shanghai Hesen Electric Co., Ltd. (Shanghai, China). Deionized water used in the experiment was produced by an ultrapure water machine. All reagents were analytical grade and were used directly without further purification.

### 2.2. Pretreatments of Multi-Walled Carbon Nanotubes (MWCNTs)

The purchased multi-walled carbon nanotubes (MWCNT, Tianjin Crystal New Material Technology Co., Ltd.) were functionalized by sonicating them in a 3:1 *v*/*v* solution of sulfuric acid (98%) and nitric acid (70%) at room temperature for 24 h to introduce hydrophilic functional groups (−OH, −COOH) on their surface [30,31,32]. Subsequently, the multi-walled carbon nanotubes were repeatedly washed with 30 mL DI water three times and dried at room temperature for later use.

### 2.3. Synthesis of Multi-Walled Carbon Nanotube Material with Cobalt Sites

**Figure 1:**
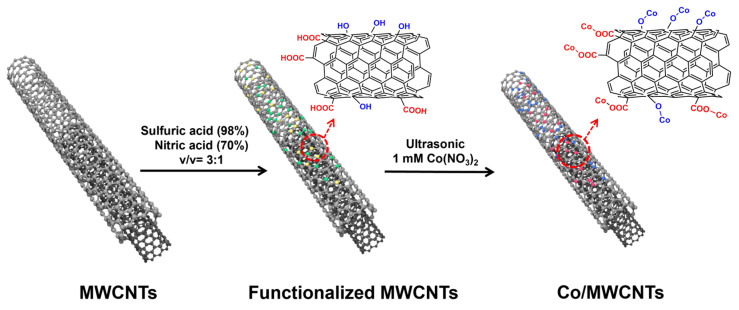
Synthesis schematic of Co/MWCNTs electrocatalyst.

The synthesis diagram of multi-walled carbon nanotube material with cobalt sites is shown in Figure 1, which includes three steps. Firstly, 10 mg of functionalized carbon nanotubes was suspended in 30 mL of deionized water in a beaker. Subsequently, 1 mmol of cobalt nitrate (0.18294 g) was added to the aqueous mixture. The mixture was then stirred at room temperature for 20 min and treated in an ultrasonic bath for 15 min to fully adsorb Co^2+^ onto the hydrophilic groups of the MWCNT surface via the electrostatic adsorption effect. The Co-adsorbed material was then separated using a centrifuge (6500 r/min for 4 min). After decanting the supernatant, the solid product was washed twice with 30 mL DI water using the same centrifugal parameters. The washed precipitate was dried in a forced-air drying oven at 60 °C for 2 h. The obtained material was named Co/MWCNTs. In contrast, Co/carbon powder was synthesized via the same procedure as that of Co/MWCNTs, except the carbon powder was used.

### 2.4. Fabrication of Working Electrode

The hydrophobic carbon paper was cut into 2 cm by 0.5 cm rectangular pieces, which were then subsequently treated with ultrasound in DI water and ethanol for 10 min. Then, the treated carbon tape pieces were dried in a blast drying oven at 60 °C for 25 min. The working electrode was constructed by evenly coating the glass bottle wall with the prepared ink, which contained well-mixed mixtures of catalyst (4 mg), isopropanol (360 μL), DI water (120 μL), and Nafion solution (20 μL). A pipette gun was used to obtain catalyst ink at a rate of 5 μL of 30 μL, which was applied to a 0.25 cm^2^ (0.5 cm × 0.5 cm) piece of carbon paper. Other parts of the carbon paper were then sealed with hot glue to obtain the electrode.

### 2.5. Characterization

Scanning electron microscope (SEM) images and energy dispersive X-ray spectroscopy (EDS) mapping were collected on the Thermo Scientific Apreo 2S (5 kV) (New York, NY, USA). Transmission electron microscopy (TEM) images were obtained using the American FEI Tecnai G2 F20 (200 kV) (Hillsborough County, OR, USA). X-ray diffraction (XRD) was performed using Rigaku Ultima IV X-ray diffractometer operating at 40 mA and 40 kV using Cu tube radiation (λ = 1.5418 Å), scanning at 2°/min from 10° to 80°. X-ray photoelectron spectroscopy was obtained through a Thermo Scientific K-Alpha spectrometer (New York, NY, USA) with monochromatic Al Kα radiation (1486.6 eV). All ultraviolet–visible (UV-Vis) absorption spectra experiments were conducted on a Hitachi UV-1900i spectrophotometer with the wavelength ranging from 500 to 800 nm at a scan rate of 600 nm min^−1^. 

### 2.6. Electrochemical Measurements

Electrochemical measurements were carried out with a CHI 760e electrochemical workstation (Shanghai, China) using a three-electrode system with a saturated calomel reference electrode. The prepared carbon paper with catalysts (0.5 cm × 0.5 cm) was used as the working electrode and the graphite rod was employed as the counter electrode. Linear sweep voltammograms (LSVs) were recorded from 0 V to −0.4 V versus RHE at a scanning rate of 10 mV s^−1^ in a single-chamber cell with a volume of 50 mL. All other tests were conducted in an H-type electrochemical cell with Nafion 115 membrane (Shanghai, China) as a separator. The cathodic section of the H-cell contained 40 mL of 1 M KOH and 0.1 M KNO_3_^−^ solution, degassed with Ar flow before tests. Linear voltammograms were recorded with a scanning rate of 20 mV s^−1^ from 0.14 to –0.46 V (vs. RHE). Unless otherwise stated, all voltages mentioned are converted to the reversible hydrogen electrode (RHE) according to the Nernst equation: E_RHE_ = E_applied_ + 0.241 + 0.059 × pH. To calculate the yield of NH_3_ and Faradaic efficiency (FE), chronoamperometry (i-t) was conducted at different potentials for 0.5 h.

### 2.7. Product Analysis and Detection

The detection of ammonia was carried out using the indophenol blue method [33,34]. This involved extracting 50 μL of electrolyte from the cathode battery and diluting it to 1 milliliter with deionized water. To this diluted sample, 1 mL of a 1 M NaOH solution containing 5% sodium citrate and 5% salicylic acid was added. Subsequently, NaClO (0.5 mL, 0.05 M) and Na_2_[Fe(NO) (CN)_5_] (0.1 mL, 1 wt%) were added to the above solution. After 2 h of chromogenic reaction, the mixture was measured by UV-vis absorption spectroscopy (500–800 nm). The absorbance at 652 nm is attributed to NH_3_ generated in the target electrolyte. To quantify the ammonia, a calibration curve was constructed using six different concentrations of (NH_4_)_2_SO_4_ solutions, ranging from 0.2 mg/mL to 6 mg/mL.

The yield rate (Y. R.) of NH_3_ was calculated by Equation (6):

YR_(NH3)_ = (C_NH3_ × V)/(t × A) (6)

The Faraday efficiency of NH_3_ is defined by comparing the charge consumed by NH_3_ generation with the total charge passing through the electrode, as described by Equation (7):

FE_(NH3)_ = (8 × F × C_NH3_ × V × 10^−6^)/(17 × Q) × 100% (7)

where C_NH3_ refers to the mass concentration (μg mL^−1^) of NH_3_ calculated from the UV-Vis curve; V represents the volume of cathode electrolyte (30 mL); t is the electrolysis time (0.5 h); F is the Faraday constant (96,485 C mol^−1^); and Q represents the total charge collected, which is integrated from the I-t curve. Under the same conditions, the experiment was conducted twice, with each potential measured twice to ensure the accuracy of the measurement.

## 3. Results and Discussions

### 3.1. Structural and Composition Characterization

**Figure 2:**
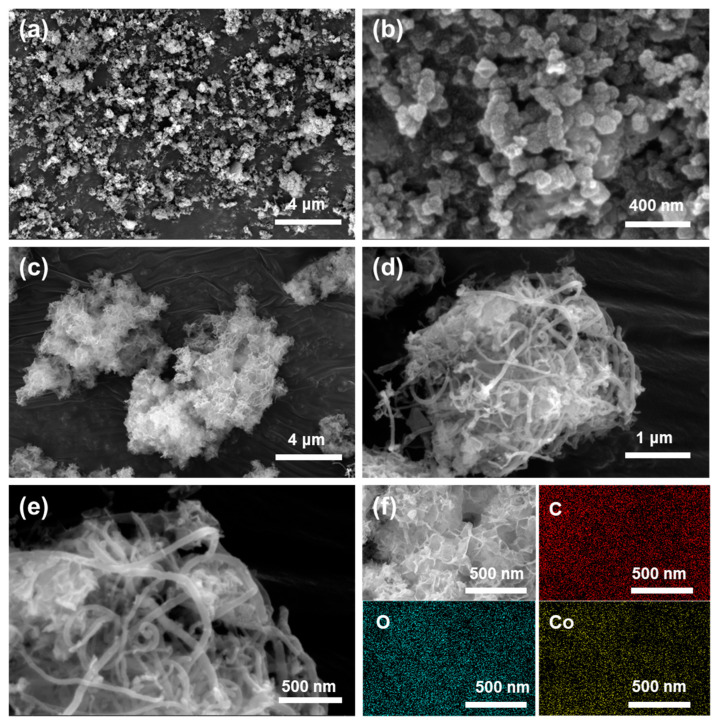
SEM images of catalysts. (**a**,**b**) SEM images of Co/carbon powder. (**c**–**e**) SEM images of Co/MWCNTs. (**f**) EDS element mapping images of Co/MWCNTs.

The morphological features of catalysts were investigated via scanning electron microscopy (SEM). As shown in Figure 2a,b, the surface morphology of the catalyst, employing carbon powder as a carrier, shows a nanoparticle granular aggregation. In contrast, Co/MWCNTs display a more open and porous surface structure, characterized by MWCNTs interweaving together (Figure 2c,d). The high-resolution SEM image of Co/MWCNTs shows the absence of obvious granular active sites on the surface of carbon nanotubes, suggesting that the cobalt active sites might be adhered to the interior of the nanotubes (Figure 2e). Elemental mapping images in Figure 2f further verified the uniform distribution of Co, O, and C elements on carbon nanotubes, indicating that the cobalt active sites in Co/MWCNTs are likely present in the form of cobalt oxides.

**Figure 3:**
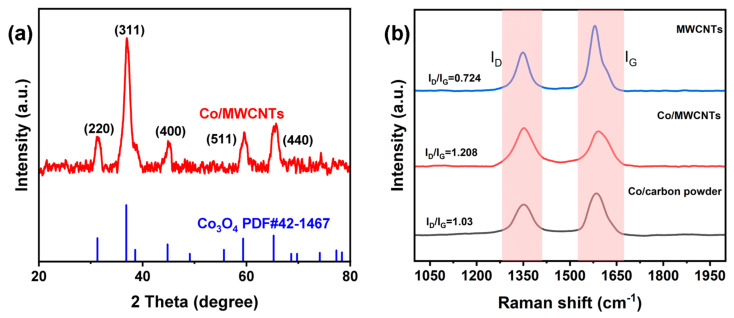
(**a**) XRD pattern of Co/MWCNTs. (**b**) Raman spectra of materials.

To confirm the composition of the catalyst, X-ray diffraction (XRD) tests were performed. As shown in Figure 3a, the diffraction peaks of Co/MWCNTs matched well with the standard card (Co_3_O_4_ PDF#42-1467), confirming that the cobalt species in the catalyst is indeed Co_3_O_4_. The pattern of Co/MWCNTs shows peaks at 2θ = 31.5, 36.7, 44.8, 59.4, and 65.4° indexed to the (220), (311), (400), (511), and (440) planes of a typical cubic Co_3_O_4_ phase (PDF#42-1467), respectively. The Raman spectroscopy results (Figure 3b) of Co/MWCNTs, Co/carbon powder, and MWCNTs all display two well-defined peaks positioned at 1350 and 1582 cm^−1^, which correspond to the disordered carbon (D band) and graphitic carbon (G band), respectively. The D band reflects sp^3^ defects in carbon, such as amorphous carbon layers and edges, in graphene, whereas the G band reflects the E_2g_ vibration of sp^2^-hybridized graphitized carbon atoms. The ratio of the peak areas of the D band to the G band (I_D_/I_G_) is usually used to evaluate the degree of defects in a graphitic structure [35,36]. The I_D_/I_G_ values of MWCNTs, Co/MWCNTs, and Co/carbon powder were found to be 0.728, 1.208, and 1.03, respectively. The highest I_D_/I_G_ value for Co/MWCNTs suggests a greater number of defects in its structure, which could imply more active sites [37].

**Figure 4:**
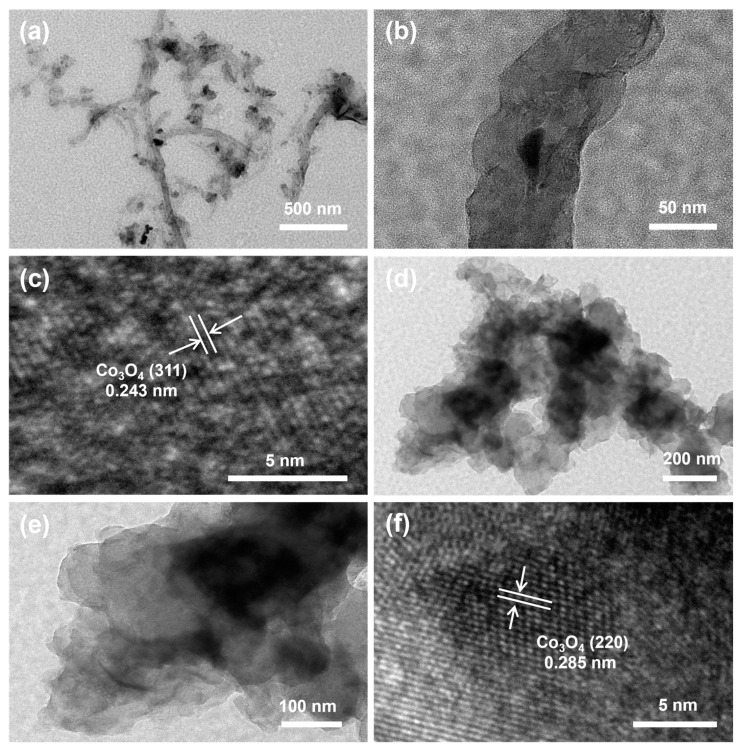
TEM images of catalysts. (**a**,**b**) TEM images of Co/MWCNTs. (**c**) High-resolution TEM image of Co/MWCNTs. (**d**,**e**) TEM images of Co/carbon powder. (**f**) High-resolution TEM image of Co/carbon powder.

Transmission electron microscopy (TEM) of Co/MWCNTs and Co/carbon powder was employed to examine the microstructure of Co/MWCNTs and the distribution of cobalt oxides on MWCNTs. It can be observed from Figure 4a,b that the MWCNTs exhibit a one-dimensional hollow nanotube structure loaded with nanoparticles. A large proportion of nanoparticles appear in lighter black, indicating that they may be inside carbon nanotubes. The high-resolution TEM (HRTEM) image demonstrates the lattice fringe spacing of 0.243 nm of the nanoparticles in Co/MWCNTs, which corresponds to the crystal plane distance of the (311) plane in the Co_3_O_4_ phase (Figure 4c). The adhesion of Co_3_O_4_ active sites inside the nanotubes may be due to the confinement effect of nanotubes, which can be attributed to the small matrix size of the MWCNTs to the nanometer scale (with an average diameter of about 20 nm) [38,39,40,41]. This not only anchors the cobalt active sites but also confines reactant molecules, such as NO_3_^−^ and NO_2_^−^, within the MWCNTs, thus facilitating the reaction. Figure 4d,e show the TEM images of Co/carbon powder. The Co/carbon powder presents larger nanoparticles, predominantly distributed within the carbon powder. The HRTEM image in Figure 4f indicates a lattice spacing of 0.285 nm, corresponding to the (220) plane of Co_3_O_4_. This observation suggests that the performance difference between Co/carbon powder and Co/MWCNTs could largely be attributed to the carrier, implying distinct mechanisms of action for the two materials.

**Figure 5:**
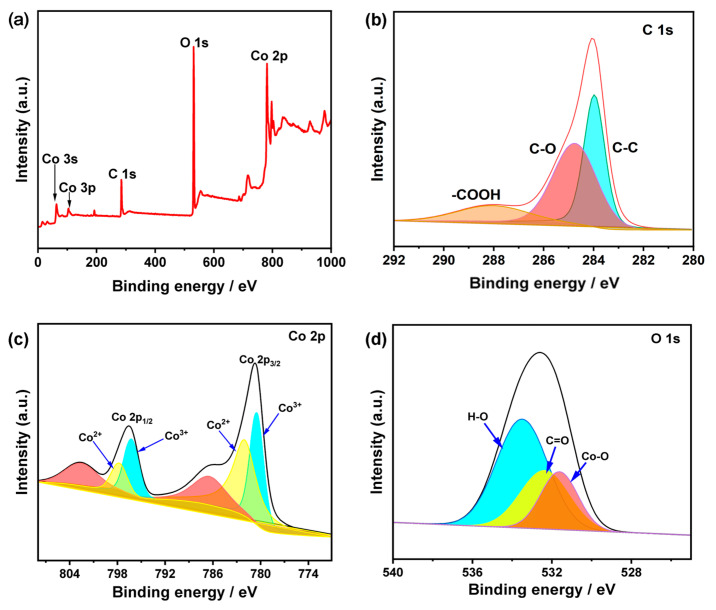
Surface compositional analyses of the Co/MWCNTs. (**a**) XPS survey spectrum of Co/MWCNTs. (**b**) C 1s and (**c**) Co 2p XPS spectra of Co/MWCNTs. (**d**) O 1s XPS spectrum of Co/MWCNTs.

X-ray photoelectron spectroscopy (XPS) was employed to further investigate the surface elemental compositions and electronic structure of Co/MWCNTs. The XPS survey spectrum (Figure 5a) confirms the existence of C, O, and Co elements in Co/MWCNTs. The high-resolution C 1s spectrum of Co/MWCNTs can be deconvoluted into three sub-bands (Figure 5b), and those at ≈284.1, 285.3, and 288.4 eV are respectively assigned to C-C, C-O, and C=O [42]. Figure 5c presents the high-resolution spectrum of Co 2p in Co/MWCNTs. The Co 2p spectrum contains two pairs of spin-orbit doublets and a pair of associated shake-up satellites. Specifically, the characteristic peaks at binding energies of 780.7 and 796.5 eV are related to Co^3+^ 2p_3/2_ and Co^3+^ 2p_1/2_, respectively, while the peaks at 782.3 and 797.8 eV belong to Co^2+^ 2p_3/2_ and Co^2+^ 2p_1/2_, implying the existence of mixed valence states of Co in Co/MWCNTs [43,44,45]. The O 1s spectra of Co/MWCNTs show three sub-bands with the binding energies at 531.2, 532.8, and 533.8 eV, which correspond to Co–O, C=O, and H–O, respectively [46]. The XPS results indicate that hydrophilic groups such as hydroxyl and carboxyl groups were successfully introduced onto the surface of MWCNTs. This modification is likely to enhance the surface properties of the catalyst and improve its capacity to adsorb reactant.

### 3.2. Product Detection and Electrochemical Properties

**Figure 6:**
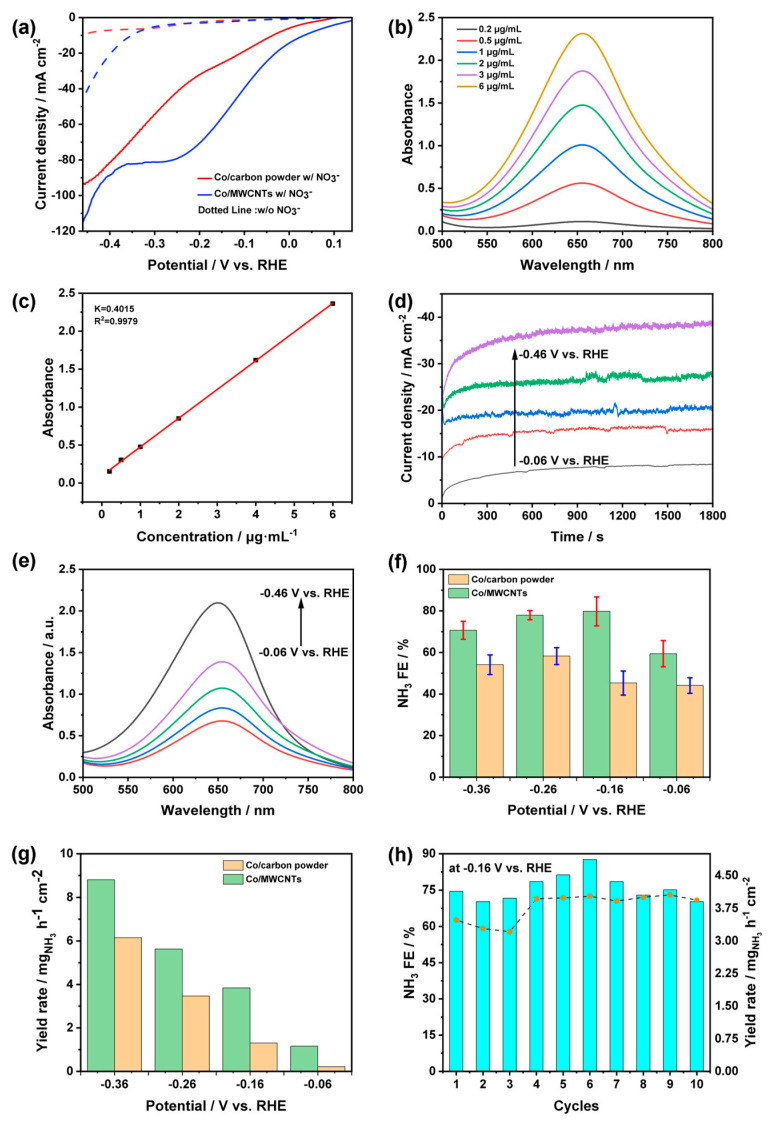
Product detection. (**a**) LSV curves of Co/MWCNTs and Co/carbon powder in 1 M KOH with or without 0.1 M KNO_3_ electrolyte (the legend of w/ and w/o corresponds to with and without NO_3_^−^). (**b**) UV-Vis spectra and (**c**) calibration curves for testing NH_3_. (**d**) I-t curves and (**e**) corresponding UV-Vis spectra of Co/MWCNTs with indophenol indicator after NitRR electrolysis at different potentials. (**f**) Faradaic efficiency of NH_3_ and (**g**) NH_3_ yield rate for Co/MWCNTs and Co/carbon powder at varying potentials. (**h**) Recycling durability test of Co/MWCNTs at −0.16 V vs. RHE (the bar chart represents yield, and the dotted line chart represents Faraday efficiency).

To evaluate the activity of the electrochemical nitrate reduction reaction (NitRR) in ammonia, linear sweep voltammetry (LSV) was conducted in an electrolyte with or without 0.1 M KNO_3_. For the LSV curve without NO_3_^−^, the Co/MWCNTs (blue dotted line) exhibited a higher current density than that of Co/carbon powder (red dotted line) at a potential of −0.36 V vs. RHE, suggesting that the HER catalytic activity of Co/MWCNTs was promoted by the MWCNTs carrier (Figure 6a). When 0.1 M NO_3_^−^ was added to the electrolyte, both catalysts exhibited a noticeable positive shift in onset potential and an increase in current density. This indicates that the thermodynamics of nitrate reduction are more favorable compared to HER and the reduction of NO_3_^−^ occurs at the Co site of both catalysts (Figure 6a). Compared to Co/carbon powder, Co/MWCNTs showed significantly higher NO_3_^−^ reduction current density, suggesting that the use of MWCNTs as a carrier enhances the catalyst’s performance. According to previous studies [38,47], it is reasonable to speculate that the Co/MWCNTs catalyst exhibits the characteristic of limiting NO_2_^−^ intermediate production within MWCNTs and hindering their diffusion, thereby promoting the conversion of NO_3_^−^ to NH_3_ and improving the selectivity of nitrate reduction to produce ammonia.

Chronoamperometry (I-t) curves were recorded to conduct product testing, and the indophenol blue spectrophotometric method was employed to determine the Faradaic efficiency (FE) and yield rate (YR) of produced ammonia. UV-Vis spectra and calibration curves for NH_3_ analysis are shown in Figure 6b and Figure 6c, respectively. The total charge (Q) passing through the Co/MWCNTs electrode was calculated from the corresponding I-t curves at various given potentials (Figure 6d). The concentration of produced ammonia in electrolytes after 30 min of electrolysis was detected using UV-Vis spectra (Figure 6e) based on the calibration curve. According to Figure 6f, the yield of NH_3_ using Co/MWCNTs was higher than that of the Co/carbon powder at various potentials. As shown in Figure 6g, Co/MWCNTs and Co/carbon powder displayed FE of 63.85% and 41.42% at the onset potential, respectively. The working electrode with MWCNTs as the carrier achieved the highest Faradaic efficiency of 84.72% at a low potential of −0.16 V versus RHE. In comparison, the Co/carbon powder showed a lower Faradaic efficiency in a wider potential range (from −0.06 to −0.36 V versus RHE) and reached the highest Faraday efficiency value of 58.75% at a negative potential of −0.26 V versus RHE. Notably, Co/MWCNTs can provide a high NH_3_ yield rate of 4.03 mg h^−1^ cm^−2^ and a high Faradaic efficiency of 84.72% at −0.16 V vs. RHE, showcasing exceptional NitRR performance at lower potential, comparable to that of recently reported transition metal electrocatalysts (Table 1). These results demonstrated that multi-walled carbon nanotubes effectively enhance the NitRR activity of Co_3_O_4_. The stability of Co/MWCNTs was studied by operating ten successive cycles of electrolysis on the same piece of catalyst. As shown in Figure 6h, Co/MWCNTs maintain an FE of around 75~84% and a YR of 3.15~3.75 mg h^−1^ cm^−2^ in each cycle, indicating excellent durability and consistent performance.

**Figure 7:**
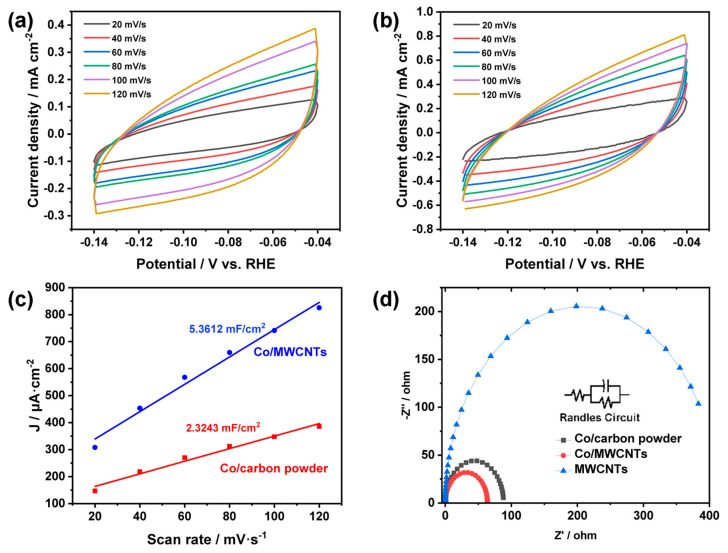
Electrochemical properties. Cyclic voltammograms of the (**a**) Co/carbon powder and (**b**) Co/MWCNTs for a series of scan rates of 20, 40, 60, 80, 100, and 120 mV s^−1^ from −0.18 to −0.08 V vs. RHE. (**c**) Electrochemical active surface area of the catalysts. (**d**) EIS patterns of the as-synthesized catalysts in 1 M KOH + 0.1 M KNO_3_ (inset: equivalent circuit).

To further elucidate the role of multi-walled carbon nanotubes in the NitRR reaction, we measured the electrochemically active surface area (ECSA) of the two working electrodes. The electrochemical double-layer capacitance (C_dl_) is an effective means to estimate the actual electrochemically active surface area (ECSA) of catalysts (ECSA ∝ C_dl_) [48,49,50]. The CV method is commonly used in experiments to calculate the double-layer capacitance. Figure 7 depicts CV curves of Co/carbon powder (Figure 7a) and Co/MWCNTs (Figure 7b) monitoring the current density in the non-Faradaic region with different scan rates. The midpoint current of the tested voltage range was linearly fitted against the scan rate relative to RHE to obtain the C_dl_ values of the double-layer capacitance. As depicted in Figure 7c, the C_dl_ value of Co/MWCNTs (5.3612 mF cm^−2^) is twice that of the Co/carbon powder (2.3243 mF cm^−2^). The ECSA was calculated according to the equation ECSA = C_dl_/C_s_, where C_s_ is 40 µF cm^−2^ based on the reported value for the flat electrode in 1.0 M KOH aqueous electrolyte [51]. We have summarized the electrochemical active surface areas of the two materials in Table 2. The larger C_dl_ for Co/MWCNTs suggests a higher accessibility of active sites as a result of their porous structures, leading to the superior catalytic activity. Combined with element mapping analysis from EDS results (Table 3), it can be seen that Co/MWCNTs contain significantly more cobalt than Co/carbon powder. This indicates that the hollow nanotube structure of MWCNTs can accommodate more active sites, which is also an advantage of multi-walled carbon nanotubes as catalyst carriers.

Transient electrochemical impedance spectroscopy (EIS) was used to analyze the resistive characteristics of the cathode materials in the nitrate reduction reaction (NitRR). The Nyquist plots of Co/MWCNTs and Co/carbon powder showed different semi-circles in the high-frequency region, indicating differences in their charge transfer resistance (R_ct_) [52]. As shown in Figure 7d, the Co/MWCNTs exhibit a smaller Rct compared with Co/carbon powder and MWCNTs, indicating the fast and efficient charge transfer during the NitRR catalytic process in alkaline electrolytes. The lower R_ct_ of Co/MWCNTs can be attributed to the functionalized MWCNTs with a hollow structure and high conductivity, which are beneficial for charge transfer and ion diffusion. As confirmed by the steady-state electrochemical analysis mentioned earlier, when using MWCNTs as a carrier, their open structure increases the active surface area of the Co/MWCNT catalyst, which is beneficial to exposing abundant active sites and providing enough space for mass transfer.

**Table 1:** Comparison of the ammonia synthesis activity of Co/MWCNTs with other catalysts.

Catalysts	Electrolytes	Yield Rate/mg cm^−2^ h^−1^	Yield Rate/mmol g^−1^ h^−1^	Faradaic Efficiency (NH_3_)/%	Ref.
CoP/TiO_2_@TP	0.1 M NaOH + 0.1 M NO_3_^−^	8.50	-	95.00 (−0.3 V vs. RHE)	[53]
S-Co_3_O_4_	0.1 M Na_2_SO_4_ + 0.1 M NO_3_^−^	-	174.20	89.90 (−0.7 V vs. RHE)	[54]
Co_3_O_4_/Co	0.1 M Na_2_SO_4_ + 1 mg mL^−1^ NO_3_^−^	4.43	-	88.70 (−0.8 V vs. RHE)	[55]
Cu_x_Co_y_HTP	0.5 M Na_2_SO_4_ + 0.1 M NO_3_^−^	5.09	-	96.40 (−0.6 V vs. RHE)	[56]
CoB@TiO_2_/TP	0.1 M Na_2_SO_4_ + 400 ppm NO_2_^−^	3.96	-	95.20 (−0.7 V vs. RHE)	[57]
Co_3_O_4_@CNF	0.1 M NaOH + 0.1 M NO_3_^–^	-	23.40	92.70 (−0.7 V vs. RHE)	[58]
Co/MWCNTs	0.1 M KOH + 0.1 M NO_3_^−^	4.03	-	84.72 (−0.1 V vs. RHE)	This work

**Table 2:** Electrochemical active surface area of the two materials.

**ECSA** **(cm^2^)**	**Co/Carbon Powder**	**Co/MWCNTs**
	58.1075	134.03

**Table 3:** The element content of materials measured by SEM-EDX.

Materials	Carbon (wt%)	Oxygen (wt%)	Cobalt (wt%)	Ratio of Elements	Total (%)
Co/MWCNTs	40.20	20.97	38.80	2:1:2	99.97
Co/carbon powder	90.17	5.58	4.25	16:1:1	100
MWCNTs	83.01	8.42	-	-	91.43

**Figure 8:**
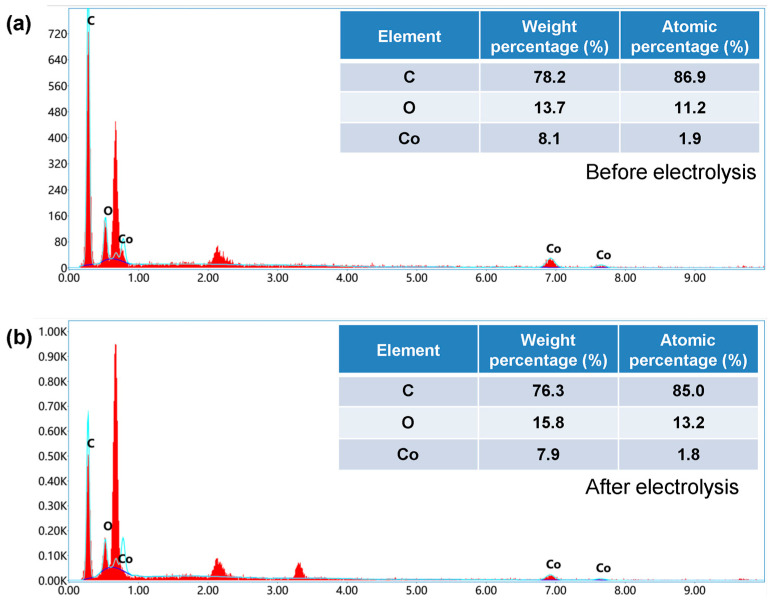
The limiting effects of MWCNTs. (**a**) Element content before electrochemical performance testing of Co/MWCNTs. (**b**) Element content after electrochemical performance testing of Co/MWCNTs.

Finally, we analyzed the element content of the material before and after testing. The element content of the same electrode before and after electrochemical performance testing was analyzed using EDS spectroscopy. The test results are shown in Figure 8a,b. From the results, the content of Co element did not show a significant decrease, indicating that MWCNTs effectively limited the exfoliation of Co species.

## 4. Conclusions

In summary, we functionalized multi-walled carbon nanotubes (MWCNTs) through acid pickling and utilized the functionalized MWCNTs as carriers to load cobalt oxide for an efficient NitRR. The introduction of hydrophilic groups onto the sidewalls of MWCNTs not only facilitated the electrostatic adsorption and provided a larger active surface area for loading cobalt trioxide (Co_3_O_4_), but also improved the wettability of MWCNTs, enabling better infiltration and uniform rapid permeation of the electrolyte. Additionally, the spatial confinement effect of MWCNTs suppresses the diffusion of intermediate substances and the detachment of cobalt species in the NitRR, thereby enhancing the stability of the catalyst. Notably, Co/MWCNTs can provide a high NH_3_ yield rate of 4.03 mg h^−1^ cm^−2^ and a high Faradaic efficiency of 84.72% at −0.16 V vs. RHE. Our results indicate that cost-effective multi-walled carbon nanotubes can be a promising carrier for nitrate reduction catalysts, which can effectively increase the nitrate reduction activity of transition metals. At the same time, this work also contributes to the exploration and recognition of the nanoconfinement effect in deepening the understanding of the growth process of functional nanoparticles in confined spaces.

## Data Availability

The data presented in this study are available upon request from the corresponding author.

## References

[1] Liang X., Zhu H., Yang X., Xue S., Liang Z., Ren X., Liu A., Wu G. (2022). Recent advances in designing efficient electrocatalysts for electrochemical nitrate reduction to ammonia. Small Struct..

[2] Zhang X., Wang Y., Liu C., Yu Y., Lu S., Zhang B. (2021). Recent advances in non-noble metal electrocatalysts for nitrate reduction. Chem. Eng. J..

[3] Singh N., Goldsmith B.R. (2020). Role of electrocatalysis in the remediation of water pollutants. ACS Catal..

[4] Liu J.-X., Richards D., Singh N., Goldsmith B.R. (2019). Activity and selectivity trends in electrocatalytic nitrate reduction on transition metals. ACS Catal..

[5] Suryanto B.H.R., Matuszek K., Choi J., Hodgetts R.Y., Du H.-L., Bakker J.M., Kang C.S.M., Cherepanov P.V., Simonov A.N., MacFarlane D.R. (2021). Nitrogen reduction to ammonia at high efficiency and rates based on a phosphonium proton shuttle. Science.

[6] Zhang M., Zhang K., Ai X., Liang X., Zhang Q., Chen H., Zou X. (2022). Theory-guided electrocatalyst engineering: From mechanism analysis to structural design. Chin. J. Catal..

[7] Foster S.L., Bakovic S.I.P., Duda R.D., Maheshwari S., Milton R.D., Minteer S.D., Janik M.J., Renner J.N., Greenlee L.F. (2018). Catalysts for nitrogen reduction to ammonia. Nat. Catal..

[8] Hawtof R., Ghosh S., Guarr E., Xu C., Mohan Sankaran R., Renner J.N. (2019). Catalyst-free, highly selective synthesis of ammonia from nitrogen and water by a plasma electrolytic system. Sci. Adv..

[9] Liu C., Li Q., Wu C., Zhang J., Jin Y., MacFarlane D.R., Sun C. (2019). Single-boron catalysts for nitrogen reduction reaction. J. Am. Chem. Soc..

[10] Gong Z., Zhong W., He Z., Liu Q., Chen H., Zhou D., Zhang N., Kang X., Chen Y. (2022). Regulating surface oxygen species on copper (i) oxides via plasma treatment for effective reduction of nitrate to ammonia. Appl. Catal. B Environ..

[11] Rosca V., Duca M., de Groot M.T., Koper M.T.M. (2009). Nitrogen cycle electrocatalysis. Chem. Rev..

[12] Daiyan R., Tran-Phu T., Kumar P., Iputera K., Tong Z., Leverett J., Khan M.H.A., Asghar Esmailpour A., Jalili A., Lim M. (2021). Nitrate reduction to ammonium: From CuO defect engineering to waste NOx-to-NH3 economic feasibility. Energy Environ. Sci..

[13] Su J.F., Kuan W.-F., Liu H., Huang C.P. (2019). Mode of electrochemical deposition on the structure and morphology of bimetallic electrodes and its effect on nitrate reduction toward nitrogen selectivity. Appl. Catal. B Environ..

[14] Qiu W., Chen X., Liu Y., Xiao D., Wang P., Li R., Liu K., Jin Z., Li P. (2022). Confining intermediates within a catalytic nanoreactor facilitates nitrate-to-ammonia electrosynthesis. Appl. Catal. B Environ..

[15] Yang J., Zhang W.-D., Zhao H., Zou Y., Zhang Z.-Y., Liu J., Wang J., Gu Z.-G., Yan X. (2024). High-valent cobalt active sites derived from electrochemical activation of metal-organic frameworks for efficient nitrate reduction to ammonia. Appl. Catal. B Environ..

[16] Liu H., Timoshenko J., Bai L., Li Q., Rüscher M., Sun C., Roldan Cuenya B., Luo J. (2023). Low-coordination rhodium catalysts for an efficient electrochemical nitrate reduction to ammonia. ACS Catal..

[17] Li Y., Cheng C., Han S., Huang Y., Du X., Zhang B., Yu Y. (2022). Electrocatalytic reduction of low-concentration nitric oxide into ammonia over Ru nanosheets. ACS Energy Lett..

[18] Liu H., Wu X., Geng Y., Li X., Xu J. Microfluidic-oriented synthesis of enriched iridium nanodots/carbon architecture for robust electrocatalytic nitrogen fixation. Green Energy Environ..

[19] Li J., Zhan G., Yang J., Quan F., Mao C., Liu Y., Wang B., Lei F., Li L., Chan A.W.M. (2020). Efficient ammonia electrosynthesis from nitrate on strained ruthenium nanoclusters. J. Am. Chem. Soc..

[20] Wang Z., Young S.D., Goldsmith B.R., Singh N. (2021). Increasing electrocatalytic nitrate reduction activity by controlling adsorption through ptru alloying. J. Catal..

[21] Li W., Xiao C., Zhao Y., Zhao Q., Fan R., Xue J. (2016). Electrochemical reduction of high-concentrated nitrate using Ti/TiO_2_ nanotube array anode and fe cathode in dual-chamber cell. Catal. Lett..

[22] Duan W., Li G., Lei Z., Zhu T., Xue Y., Wei C., Feng C. (2019). Highly active and durable carbon electrocatalyst for nitrate reduction reaction. Water Res..

[23] Wang Z., Zhao J., Wang J., Cabrera C.R., Chen Z. (2018). A Co–N4 moiety embedded into graphene as an efficient single-atom-catalyst for no electrochemical reduction: A computational study. J. Mater. Chem. A.

[24] Li X., Xing W., Hu T., Luo K., Wang J., Tang W. (2022). Recent advances in transition-metal phosphide electrocatalysts: Synthetic approach, improvement strategies and environmental applications. Coord. Chem. Rev..

[25] Guo D.-J., Jing Z.-H. (2010). A novel co-precipitation method for preparation of Pt-CeO_2_ composites on multi-walled carbon nanotubes for direct methanol fuel cells. J. Power Sources.

[26] Dilpazir S., He H., Li Z., Wang M., Lu P., Liu R., Xie Z., Gao D., Zhang G. (2018). Cobalt single atoms immobilized N-Doped carbon nanotubes for enhanced bifunctional catalysis toward oxygen reduction and oxygen evolution reactions. ACS Appl. Energy Mater..

[27] Ratso S., Kruusenberg I., Joost U., Saar R., Tammeveski K. (2016). Enhanced oxygen reduction reaction activity of nitrogen-doped graphene/multi-walled carbon nanotube catalysts in alkaline media. Int. J. Hydrogen Energy.

[28] Ratso S., Kruusenberg I., Vikkisk M., Joost U., Shulga E., Kink I., Kallio T., Tammeveski K. (2014). Highly active nitrogen-doped few-layer graphene/carbon nanotube composite electrocatalyst for oxygen reduction reaction in alkaline media. Carbon.

[29] Su X., Wang R., Li X., Araby S., Kuan H.-C., Naeem M., Ma J. (2022). A comparative study of polymer nanocomposites containing multi-walled carbon nanotubes and graphene nanoplatelets. Nano Mater. Sci..

[30] Zheng Y., Jiao Y., Zhu Y., Cai Q., Vasileff A., Li L.H., Han Y., Chen Y., Qiao S.-Z. (2017). Molecule-level g-C3N4 coordinated transition metals as a new class of electrocatalysts for oxygen electrode reactions. J. Am. Chem. Soc..

[31] Naqvi S.T.R., Rasheed T., Hussain D., Najam ul Haq M., Majeed S., Shafi S., Ahmed N., Nawaz R. (2020). Modification strategies for improving the solubility/dispersion of carbon nanotubes. J. Mol. Liq..

[32] Kim S.W., Kim T., Kim Y.S., Choi H.S., Lim H.J., Yang S.J., Park C.R. (2012). Surface modifications for the effective dispersion of carbon nanotubes in solvents and polymers. Carbon.

[33] Li P., Jin Z., Fang Z., Yu G. (2021). A single-site iron catalyst with preoccupied active centers that achieves selective ammonia electrosynthesis from nitrate. Energy Environ. Sci..

[34] Zhao X., Jia X., He Y., Zhang H., Zhou X., Zhang H., Zhang S., Dong Y., Hu X., Kuklin A.V. (2021). Two-dimensional BCN matrix inlaid with single-atom-Cu driven electrochemical nitrate reduction reaction to achieve sustainable industrial-grade production of ammonia. Appl. Mater. Today.

[35] Yang X., Li K., Cheng D., Pang W.-L., Lv J., Chen X., Zang H.-Y., Wu X.-L., Tan H.-Q., Wang Y.-H. (2018). Nitrogen-doped porous carbon: Highly efficient trifunctional electrocatalyst for oxygen reversible catalysis and nitrogen reduction reaction. J. Mater. Chem. A.

[36] Ni B., Chen R., Wu L., Xu X., Shi C., Sun P., Chen T. (2020). Optimized enhancement effect of sulfur in Fe–N–S Codoped Carbon Nanosheets for Efficient Oxygen Reduction Reaction. ACS Appl. Mater. Interfaces.

[37] Cai J., Zhang H., Zhang L., Xiong Y., Ouyang T., Liu Z.-Q. (2023). Hetero-anionic structure activated Co–S bonds promote oxygen electrocatalytic activity for high-efficiency zinc–air batteries. Adv. Mater..

[38] Guan L., Suenaga K., Shi Z., Gu Z., Iijima S. (2007). Polymorphic structures of iodine and their phase transition in confined nanospace. Nano Lett..

[39] Medeiros P.V.C., Marks S., Wynn J.M., Vasylenko A., Ramasse Q.M., Quigley D., Sloan J., Morris A.J. (2017). Single-atom scale structural selectivity in te nanowires encapsulated inside ultranarrow, single-walled carbon nanotubes. ACS Nano.

[40] Vasylenko A., Marks S., Wynn J.M., Medeiros P.V.C., Ramasse Q.M., Morris A.J., Sloan J., Quigley D. (2018). Electronic structure control of Sub-nanometer 1d SnTe via Nanostructuring within Single-Walled Carbon Nanotubes. ACS Nano.

[41] Zeng X., Zhang H., Zhang X., Zhang Q., Chen Y., Yu R., Moskovits M. (2022). Coupling of ultrasmall and small CoxP nanoparticles confined in porous SiO_2_ matrix for a robust oxygen evolution reaction. Nano Mater. Sci..

[42] Wan X., Du H., Tuo D., Qi X., Wang T., Wu J., Li G. (2023). Uio-66/carboxylated multiwalled carbon nanotube composites for highly efficient and stable voltammetric sensors for gatifloxacin. ACS Appl. Nano Mater..

[43] Jia R., Wang Y., Wang C., Ling Y., Yu Y., Zhang B. (2020). Boosting selective nitrate electroreduction to ammonium by constructing oxygen vacancies in TiO_2_. ACS Catal..

[44] Zhao D., Ma C., Li J., Li R., Fan X., Zhang L., Dong K., Luo Y., Zheng D., Sun S. (2022). Direct eight-electron NO^3−^-to-NH_3_ conversion: Using a Co-doped TiO_2_ nanoribbon array as a high-efficiency electrocatalyst. Inorg. Chem. Front..

[45] Wang D., Chen Z.-W., Gu K., Chen C., Liu Y., Wei X., Singh C.V., Wang S. (2023). Hexagonal cobalt nanosheets for high-performance electrocatalytic no reduction to NH3. J. Am. Chem. Soc..

[46] Li L., Wang Q., Zhang X., Fang L., Li X., Zhang W. (2020). Unique three-dimensional Co3O4@CNFs derived from zifs and bacterial cellulose as advanced anode for sodium-ion batteries. Appl. Surf. Sci..

[47] Yao F., Xia M., Zhang Q., Wu Q., Terasaki O., Gao J., Jin C. (2022). Confinement effect induced conformation change of one-dimensional phosphorus chains filled in carbon nanotubes. Carbon.

[48] Gao T., Zhou C., Zhang Y., Jin Z., Yuan H., Xiao D. (2018). Ultra-fast pyrolysis of ferrocene to form Fe/C heterostructures as robust oxygen evolution electrocatalysts. J. Mater. Chem. A.

[49] Gao T., Jin Z., Liao M., Xiao J., Yuan H., Xiao D. (2015). A trimetallic V–Co–Fe oxide nanoparticle as an efficient and stable electrocatalyst for oxygen evolution reaction. J. Mater. Chem. A.

[50] Yoon Y., Yan B., Surendranath Y. (2018). Suppressing ion transfer enables versatile measurements of electrochemical surface area for intrinsic activity comparisons. J. Am. Chem. Soc..

[51] Patil S.A., Khot A.C., Chavan V.D., Rabani I., Kim D.-K., Jung J., Im H., Shrestha N.K. Electrostatically robust CoFeOF nanosheet against chloride for green-H_2_ production in alkaline seawater electrolysis. Chem. Eng. J..

[52] Zhang H., Liu Y., Chen T., Zhang J., Zhang J., Lou X.W. (2019). Unveiling the Activity Origin of Electrocatalytic Oxygen Evolution over Isolated Ni Atoms Supported on a N-Doped Carbon Matrix. Adv. Mater..

[53] Deng Z., Ma C., Fan X., Li Z., Luo Y., Sun S., Zheng D., Liu Q., Du J., Lu Q. (2022). Construction of CoP/TiO_2_ nanoarray for enhanced electrochemical nitrate reduction to ammonia. Mater. Today Phys..

[54] Niu Z., Fan S., Li X., Yang J., Wang J., Tao Y., Chen G. (2023). Tailored electronic structure by sulfur filling oxygen vacancies boosts electrocatalytic nitrogen oxyanions reduction to ammonia. Chem. Eng. J..

[55] Zhao F., Hai G., Li X., Jiang Z., Wang H. (2023). Enhanced electrocatalytic nitrate reduction to ammonia on cobalt oxide nanosheets via multiscale defect modulation. Chem. Eng. J..

[56] Liu P., Yan J., Huang H., Song W. (2023). Cu/Co bimetallic conductive mofs: Electronic modulation for enhanced nitrate reduction to ammonia. Chem. Eng. J..

[57] Hu L., Zhao D., Liu C., Liang Y., Zheng D., Sun S., Li Q., Liu Q., Luo Y., Liao Y. (2022). Amorphous CoB nanoarray as a high-efficiency electrocatalyst for nitrite reduction to ammonia. Inorg. Chem. Front..

[58] Zhong L., Chen Q., Yin H., Chen J.S., Dong K., Sun S., Liu J., Xian H., Li T. (2023). Co_3_O_4_ nanoparticles embedded in porous carbon nanofibers enable efficient nitrate reduction to ammonia. Chem. Commun..

